# Lipid Mediators as Therapeutic Targets in Prostate Cancer

**DOI:** 10.3390/cancers18203315

**Published:** 2026-10-14

**Authors:** Pravita Balijepalli, Kathryn E. Meier

**Affiliations:** Department of Pharmaceutical Sciences, College of Pharmacy and Pharmaceutical Sciences, Washington State University, Spokane, WA 99202, USA; pravitabalijepalli@gmail.com

**Keywords:** G protein-coupled receptors, sphingosine 1-phosphate, lysophosphatidic acid, prostaglandins, thromboxanes, leukotrienes, cannabinoids, free fatty acids

## Abstract

Lipid mediators are small molecules, derived from lipid metabolism, that initiate cellular responses. In this review, the major classes of lipid mediators are considered with respect to their effects on prostate cancer cells. These classes are: sphingolipids (sphingosine 1-phosphate and ceramide), glycophospholipids (lysophosphatidic acid; LPA), eicosanoids (prostaglandins, thromboxanes, and leukotrienes), cannabinoids, and free fatty acids. Almost all of these mediators elicit effects by binding to druggable G protein-coupled receptors (GPCRs) to initiate cell proliferation, stimulate migration, and enhance survival. The exceptions are omega-3 fatty acids and endocannabinoids, which inhibit proliferation. The mediators and their receptors serve as potential targets for (1) enzyme inhibitors that block their synthesis, or (2) GPCR antagonists or agonists. These agents are in various stages of development. Progress in testing their translational potential varies between the mediator classes. Lipid mediators also show potential as diagnostic or prognostic biomarkers. Interesting mechanistic aspects have been identified in the course of studying lipid mediator responses in prostate cancer cells. These include positive cross-talk between LPA receptors and EGF receptors, and negative cross-talk between LPA receptors and free fatty acid (FFA) receptors. Together, these findings suggest new strategies for modulating prostate cancer growth and survival pathways in a therapeutic direction.

## Simple Summary

This article reviews the roles of lipid mediators as potential therapeutic targets in prostate cancer. Lipid mediators are derived from more complex cellular phospholipids and some come from the diet. The major mediators discussed are sphingosine 1-phosphate, lysophosphatidic acid, prostaglandin E_2_, thromboxane A_2_, lipoxin A_4_, endocannabinoids, and omega-3 fatty acids. All activate distinct druggable G protein-coupled receptors (GPCRs). Most of these mediators stimulate prostate cancer cell proliferation, survival, and migration; exceptions are endocannabinoids and omega-3 fatty acids which are inhibitory. Lipid mediators are emerging targets in prostate cancer; most work has been done in cell culture or animal models. Pharmacologic agents that target GPCRs (to either activate or block the receptor) or metabolic enzymes (to inhibit mediator synthesis) are still under development, with clinical trials just beginning. These novel drugs have the potential to augment the effects of chemotherapeutic agents, and/or to maintain a physiologic environment that discourages prostate cancer progression.

## 1. Introduction

Historically, studies of endocrine, paracrine, and autocrine mediators in cancer have focused on water-soluble small molecules, or on peptides. This is largely for practical reasons, as these molecules are relatively easy to detect and measure (e.g., in plasma), and their actions have been extensively examined. In contrast, lipid molecules have been more difficult to quantify, are often labile, have limited water solubility, and can arise from multiple complex enzymatic pathways. Nonetheless, the technical challenges have been gradually overcome. This progress in analytics, along with identification of the receptors activated by lipid mediators, has provided us with insights into the roles of these interesting and important molecules in cancer and in multiple other conditions.

Lipid mediators have been of particular interest in prostate cancer. Whether this is because lipid mediators are particularly important in this disease, because there has been funding specifically directed to prostate cancer, or because of the urgent need for new approaches to treat prostate cancer, is interesting to consider but not critical. Whatever the motivation, prostate cancer cells have served as excellent and clinically relevant model systems in which investigators can explore the roles of particular lipid mediators as therapeutic targets. The emergence of small molecule agonist and antagonist ligands for the receptors recognizing these mediators, which are largely G protein-coupled receptors (GPCRs), has facilitated this productive exploration.

The objective of this review was to provide an overall view of the lipid mediators being examined in prostate cancer, and the pre-clinical data suggesting potential therapeutic targets. This article is organized by chemical classes of lipid mediators, beginning with those most extensively studied. The major classes discussed are glycerophospholipids (lysophosphatidic acid), sphingolipids (sphingosine 1-phosphate and ceramides), eicosanoids (prostaglandins, thromboxanes, and leukotrienes), cannabinoids, and free fatty acids. All of these mediators are agonists for G protein-coupled receptors (GPCRs). Advances in drug development and results from preliminary clinical trials are discussed. Since the scope is broad, and the number of published reports is variable between the different lipid mediators, this review is designed to be representative, but not comprehensive, in covering the data that is currently available. Within each section, the literature is generally presented in chronological order.

## 2. Lipid Mediators by Class

### 2.1. Sphingolipids

Sphingolipids comprise a complex family of abundant membrane lipids. Sphingolipids that serve as lipid mediators include sphingosine 1-phosphate (S1P) and ceramides. Pathways for sphingolipid synthesis and metabolism have been comprehensively reviewed [1]. Of most relevance to cell signaling, S1P is produced by phosphorylation of sphingosine by sphingosine kinases (SPHK) 1 and 2. S1P initiates diverse cellular responses including proliferation and migration, and tissue responses including inflammation and tumor metastasis. The pleiotropic effects of S1P in cancer cells have been reviewed [2]; the authors mention that S1P activates signaling pathways that include Ras/ERK, RhoA, NLRP3/IL-1β, PI3K/AKT, PI3K/Rac, STAT3, PLC. COX-2, and MMP-9.

Ceramide can be produced by multiple pathways, but for signaling purposes ceramides are generally generated by the action of sphingomyelinases on sphingomyelin, or by ceramide synthase which attaches a sphingoid base to a fatty acid. Ceramides are degraded by ceramidases. Notably, ceramide can be metabolized to sphingosine, which can be phosphorylated to produce S1P as noted above. Thus, S1P and ceramide are intertwined within sphingolipid metabolism pathways.

S1P serves as an agonist for GPCR S1P receptors designated as S1PR1-5; some of these receptors are closely related (by sequence and structure) to lysophosphatidic acid (LPA) receptors [3]. By activating S1PRs, which are variably coupled to typical GPCR downstream pathways, S1P generally acts as a growth factor to stimulate the proliferation of cancer cells. The responses mediated by the different S1PRs are slightly different but not generally contradictory, so the experimental focus is typically on those receptors that are most highly expressed in a given cell or tissue. Ceramide exerts cellular actions through multiple mechanisms, including effects on membrane organization and binding to diverse proteins including a variety of GPCRs [4]. With respect to cancer, ceramides generally exert a negative effect and can induce apoptosis. However, as noted above, ceramide can serve as a substrate for the production of S1P. Thus, there is considerable complexity to be considered here.

#### 2.1.1. Sphingosine 1-Phosphate

In an early study, investigators examined the expression of SPHK and S1P receptors in human prostate cancer cell lines [5]. They found that SPHK1, S1PR1, and S1PR3 were more highly expressed in PC-3 and Du145 human prostate cancer cells (androgen-independent) than in LNCaP cells (androgen-dependent). An SPHK1 pharmacologic inhibitor or SPHK1 knockdown reduced proliferation of PC-3 cells; this effect was attenuated by addition of exogenous S1P. The same study showed that the cancer chemotherapeutic agent camptothecin increased expression of SPHK1, S1PR1, and S1PR3 in PC-3 cells. Our group also examined the expression of S1P receptors in PC-3, Du145, and LNCaP cells [6]. We found that all three cell lines expressed mRNA for S1P receptors 1, 2, and 3; S1PR1 and S1PR3 were most highly expressed in PC-3 cells. S1PR5 transcript and protein was expressed in all three cell lines, with the highest level in LNCaP. S1P induced cell proliferation in PC-3 and Du145, but not LNCaP. S1P activated the protein kinases Erk, Akt, and FAK in PC-3 and Du145 cells but not in LNCaP cells. We subsequently reported that S1P induces CCN1, a matricellular protein, in PC-3 cells [7]. In contrast to these results, another group found that S1P increased intracellular calcium levels in PC-3 and Du145 cells, but rather than stimulating proliferation, S1P induced apoptosis [8]. The reasons for the discrepancy are unknown, but could reflect differences between cell strains or culture conditions. Others observed that S1P induced autophagy of PC-3 cells via S1PR5 [9].

Researchers have explored the role of SPHK1 in prostate cancer. One group found that short-term androgen deprivation of PC-3 cells resulted in a transient inhibition of SPHK1 activity that was accompanied by decreased proliferation [10]. Long-term androgen deprivation, which eventually resulted in a neuroendocrine phenotype, increased SPHK1 activity and expression. Exploring the idea of SPHK1 as a therapeutic target that enhances tumor cell survival, one team found that an SPHK1 pharmacologic inhibitor sensitized LNCaP and PC-3 cells to the growth inhibitory effects of both camptothecin and docetaxel [11]. In a rodent xenograft model, the SPHK1 inhibitor acted synergistically with camptothecin to reduce the growth of PC-3 cell tumors. SPHK inhibitors have also shown efficacy in overcoming enzalutamide resistance [12].

Subsequent publications have further delineated signal transduction pathways involved in S1P action. For example, one study showed that S1P activated STAT3 in Du145 cells but not in LNCaP or PC-3 cells [13]. This response was inhibited by a MEK inhibitor or by pertussis toxin. S1P induced migration and invasion in this report. This team reported that S1PR2 and S1PR3 were expressed more highly than S1PR1 in Du145 cells; an S1PR2 antagonist and a mixed S1PR1/S1PR3 antagonist reduced S1P-induced Stat3 phosphorylation and cell migration. Another group found that S1P activated S1PR5 to induce ER stress and autophagy in PC-3 cells [14]. This response is cytoprotective. Others evaluated interactions between osteoclasts and prostate cancer cells in the tumor microenvironment, using cell line models [15]. They found that co-culture of the two cell types, or addition of conditioned medium from osteoclast-like cells to prostate cancer cells, stimulated cancer cell proliferation in a S1P-dependent manner. Using a variety of strategies, the investigators found that SPHK1 and the S1PR1 receptor were involved in the paracrine pathway. This very extensive study also examined the role of Spns2, an S1P transporter.

Further investigations have delved further into the potential clinical significance of S1P in prostate cancer. One group demonstrated that sphingosine lyase (SPL), the enzyme that degrades and inactivates S1P, is over-expressed in human prostate cancer tissue samples as compared to adjacent non-cancer tissue [16]. Knockdown of SPL enhanced sensitivity to irradiation or chemotherapy; a combination of SPL and SPHK1 silencing was even more effective in this regard. The same group later reported that an S1P-neutralizing antibody reduces intratumoral blood perfusion, and sensitizes animals to chemotherapy, in a rodent xenograft model of prostate cancer [17]. A recent report showed that IGFBP3 is up-regulated in enzalutamide-resistant prostate cancer cell lines and in prostate cancer samples from enzalutamide-treated human patients [18]. IGFBP3 was shown to activate SPHK1 and increase S1P levels. An SPHK1 inhibitor enhanced enzalutamide sensitivity. Along similar lines, another group found that doclataxel and camptothecin inhibited SPHK1 in PC-3 and LNCaP cells, increasing the ratio of ceramide to S1P [19]. These investigators showed that silencing of SPHK1 in PC-3 cells resulted in increased tumor size and doclataxel resistance in a mouse orthotopic tumor model. Others also reported that the SPHK1/2 inhibitor SKI-178 inhibited proliferation of prostate cancer cell lines in culture, and also suppressed PC-3 tumor growth in a rodent xenograft model [20]. Together, these findings confirm that S1P is important for prostate cancer growth. Accordingly, a phase II clinical trial was conducted to test whether opaganib, an inhibitor of SPHK2 and dihydroceramide desaturase, had an anti-cancer effect in patients with metastatic castration-resistant prostate cancer [21]. This study followed a phase I trial demonstrating safety and showing preliminary anti-cancer effects. While the primary objective of 30% disease control was not met in the phase II study, a subset of patients showed a positive response to opaganib. This study requires further follow-up.

Recently, the expression of S1P receptors was further addressed [22]. The authors concluded that S1P receptors are highly expressed in human prostate. S1PR1, S1PR2, and S1PR3 were most prevalent and were localized to epithelial/stromal compartments. S1PR1 and S1PR3 mediated enhanced proliferation in cell lines via activating Erk and Akt, stimulated cell contraction via RhoA/ROCK, and suppressed inflammation via STAT3. Administration of S1P and S1PR1/3 agonists to rats caused prostate enlargement; antagonists inhibited testosterone-induced benign prostatic hypertrophy (BPH).

Additional studies have examined the role and biomarker potential of circulating S1P in prostate cancer. In 2012, researchers measured plasma levels of S1P in human patients using an enzymatic assay available at the time [23]. They found that circulating S1P was lower in prostate cancer patients than in healthy or BPH patients, and that S1P levels were inversely correlated with cancer progression, metastasis, and mortality. SPHK1 activity in erythrocytes were higher in healthy patients than in cancer patients. In 2017, another group used a lipidomic approach to identify a “3-lipid signature” (3LS) consisting of specific species of ceramide, sphingomyelin, and phosphatidylcholine that were prognostic of castration-resistant prostate cancer [24]. In 2026, others further examined the effects of 3LS plasma on prostate cancer cells [25]. They found that 3LS plasma from cancer patients increased the viability of PC-3 and C4-2B cell lines. These investigators implicated S1P as the primary factor responsible, based on the interfering effect of an SPHK1 inhibitor.

In summary, the role of S1P in prostate cancer has been examined from multiple aspects, including enzymes involved in S1P synthesis and degradation, S1P transporters, and S1P receptors. The interrelationships here are as follows. Inhibition of SPHK reduces the synthesis of S1P, depriving prostate cancer cells of a lipid growth factor. SPHK inhibitors also shift the balance toward ceramide, a pro-apoptotic mediator (see below). In the absence of an SPHK inhibitor, increased ceramide results in increased S1P. S1P transporters facilitate the entry of extracellular S1P into cells, where it can activate S1P receptors intracellularly. SPL degrades S1P, and is overexpressed in prostate cancer. SPL knockdown enhances cell death via a mechanism that is not clear. Some of these seemingly contradictory findings may have to do with effects of the interventions on S1P/ceramide balance. Multiple S1P receptors are expressed in prostate cancer cell lines and tissues, with S1PR1 and S1PR3 emerging as most highly expressed and likely most relevant with respect to tumor growth. As therapeutic targets, S1P receptors have the advantage of targeting specific GPCRs and their downstream signaling, rather than perturbing the overall S1P/ceramide balance. The potential for S1P to serve as a diagnostic or prognostic marker in prostate cancer has been explored. The literature on this subject is robust and evolving, and was recently reviewed with an emphasis on the therapeutic potential of SPHK inhibitors [3,26]. These articles list different classes of anti-S1P agents and mention clinical trials that were ongoing at the time. Several S1PR1 receptor antagonists are already approved as immune modulating agents for the treatment of multiple sclerosis. S1PR-targeted drug development is continuing with the goal of improving efficacy, selectivity, and therapeutic index [27]. One consideration in cancer therapy is the immunosuppressive action of S1PR1 antagonists, which could be counterproductive to tumor immunotherapy.

#### 2.1.2. Ceramides

In 2006, a collaborative team showed that the viral protein apoptin, which induces apoptosis in tumor cells, increased ceramide levels in PC-3, Du245, and LNCaP cells [28]. They also observed that many primary human tumors over-expressed acid ceramidase (AC), which breaks down ceramide. Overexpression of AC reduced sensitivity to apoptin, while a pharmacologic AC inhibitor increased sensitivity to apoptin-induced apoptosis in both cell lines and Du145 rodent xenografts.

Lin and colleagues have pursued an alternative aspect of the role of ceramide in prostate cancer. These investigators have noted that elevated circulating ceramide is associated with poor clinical outcomes. This may be due to conversion of ceramide to the pro-survival mediator S1P. The investigators speculated that, since statins have a beneficial effect in prostate cancer (independent of cholesterol-lowering effects), and also lower circulating ceramide levels, and since higher ceramide levels are associated with higher risk of progression to androgen-resistant prostate cancer [29], then targeting the sphingolipid “rheostat” via SPHK could be a viable therapeutic strategy. This concept was explored in a publication showing that SPHK inhibitors can enhance prostate cancer cell death induced by enzalutamide and other androgen receptor signaling inhibitors [12].

Additional work is warranted to distinguish the opposing direct apoptotic roles of ceramides on prostate cancer cells, versus their indirect effects in increasing S1P levels.

### 2.2. Glycerophospholipids

Glycerol-based phospholipids are the major phospholipid constituents of plasma membranes. With respect to prostate cancer, the glycerophospholipid metabolite that has been of the most interest is lysophosphatidic acid (LPA), which is generated largely from the abundant phospholipid, phosphatidylcholine. The major pathway for synthesis of extracellular LPA is via autotaxin, a secreted and inducible form of lysophospholipase D that converts lyso-phosphatidylcholine to LPA [30,31]. There are also membrane-localized forms of lysophospholipase D that can generate LPA within cells [32]. Phosphatidic acid generated intracellularly by phospholipase D or other routes can be converted to LPA by phospholipase A_2_. Acylglyerol kinase, a mitochondrial enzyme, can generate LPA from monoacylglycerol. LPA and autotaxin are present in seminal fluid, where LPA can be degraded by prostatic acid phosphatase [33].

The biological effects of LPA are largely mediated by a family of GPCRs termed LPAR1-6. There are six known LPA receptors, which are coupled to distinct and overlapping signaling pathways [34,35]. These widely expressed GPCRs are distinct in their downstream signaling pathways, which encompass all known GPCR-activated responses [36]. Most cells express more than one type of LPA receptor. Through some of its receptors (e.g., LPAR1), LPA is particularly efficacious at activating the small GTPase Rho, which regulates the actin cytoskeleton and cell motility. In cancer cells, LPA generally promotes cell proliferation and migration.

#### Lysophosphatidic Acid

Lysophosphatidic acid (LPA) refers to a class of simple phospholipids that are largely derived from phosphatidylcholine. The different species of LPA have different fatty acid substituents. The species that is typically used experimentally is 18:1(oleoyl)-LPA, which is particularly efficacious at activating certain LPA receptors (e.g., LPAR1). It should be noted that multiple LPA species with different activities exist in human plasma, but that 18:1 LPA is generally referred to as simply “LPA”.

LPA is nearly ubiquitous in the human body, is present in plasma, and exerts effects on a wide range of tissues. In cancer cells, LPA generally promotes proliferation [37]. Since one of the prominent downstream pathways utilized by LPA receptors entails activation of Rho, LPA is particularly effective at stimulating cell migration. Roles for LPA in prostate cancer progression have been reviewed [38].

In 1998, our group reported that 18:1-LPA stimulated proliferation and phospholipase D (PLD) activity in prostate cancer cells [39]. We had been studying activation of PLD, which generates phosphatidic acid, and became interested in LPA as its potential downstream metabolite. Subsequently, we examined the expression of LPA receptors in prostate cancer cell lines [40]. Further work showed that prostate cancer cells can produce LPA in response to stimulation of another GPCR, and can respond to that LPA; this raised the possibility that LPA is an autocrine mediator [41]. Later studies examined LPA- and S1P-induced signaling pathways in more detail in prostate cancer cell lines [5], and showed that LPA induces calpain-mediated proteolysis of FAK in these cells [42]. Subsequent examinations led to the conclusion that the effects of LPA in prostate cancer cell lines are mediated largely by LPAR1 [43]. This study also revealed an interesting and surprising dependence of epidermal growth factor (EGF) response on LPAR1, in that LPAR1 antagonist or LPAR1 knockdown suppressed the response to EGF. Having previously observed that LPA induces the matricellular protein CCN1 in prostate cancer cells [44], we further explored the role of CCN1 in LPA action [7]. CCN1 was shown to contribute to LPA-induced cell adhesion. We then conducted a global proteomics analysis of the effects of LPA on PC-3 cells [45] that revealed LPA induction of several proteins involved in cell–substrate adhesion, some of which were induced in a CCN1-dependent manner. The induced proteins included thrombospondin-1, which had been previously shown to be up-regulated by LPA in PC-3 cells [46], and down-regulation of LPAR1 in response to LPA. We showed that PC-3 cells express autotaxin as well as GDE4 and GDE7 [47]. Most recently, following up on the selectivity of LPA receptors to be activated by different species of LPA, we collaborated with others to define the structural basis of the selectivity of LPAR1 for 18:2-LPA (active) versus 18:0-LPA (inactive) [48].

Substantial work in this area was accomplished by the Daaka research group, in multiple published studies. This group also examined the expression of LPARs in prostate cancer cell lines, focusing in on LPAR1 [49]. These investigators studied LPA-induced Erk activation in PC-3 cells, revealing a mechanism involving LPA transactivation of EGF receptor (EGFR) [50]. Their work established that LPA can promote survival of PC-3 cells via activation of NF-kB [51]. The group also showed that LPAR1 is the most important receptor for LPA-induced proliferation in prostate cancer cells [52].

A recent review noted that LPA has a significant role to play in castration-resistant prostate cancer by contributing to an aggressive phenotype, either bypassing other growth factors or acting in their absence [53]. Additional studies have examined the interplay between LPA receptors and EGFR. LPA initiates crucial crosstalk between LPARs and EGFR; either endogenous LPA (generated via acylglycerol kinase) or exogenous LPA transactivates EGFR to drive downstream Erk signaling [49,50,54]. Conversely, as mentioned above, LPAR1 expression/activity is reciprocally required for robust cellular responses to EGF [43]. The interesting positive cross-talk between LPAR1 and EGFR has not been fully explored mechanistically, and could lead to new therapeutic avenues. In other words, if LPAR1 is required for EGF response, then this broadens the impact of LPA receptor inhibition since EGFR is an important therapeutic target in many types of cancers. To date, most studies related to this mechanism have focused on the role of LPA (and other growth factors) in releasing ligands that can activate EGFR [49]. However, it is also possible that LPARs (and possibly other GPCRs) interact directly with EGFR in a signaling complex to promote cell growth. There is evidence for EGFR-GPCR heterodimerization for other GPCRs, including S1P1 [55,56,57]. If this is the case, than combined therapies that target LPARs and EGFR might have a synergistic anti-cancer effect.

Other observations are as follows. Studies of LPA action in cell culture typically utilize serum-starved cells, since serum contains LPA. However, serum deprivation causes autophagy. One group showed that LPA inhibits autophagy in PC-3 cells [58]. In 2009, it was reported that LPAR3 expression levels were higher in prostate tumor cells than in benign tissue; this study also examined expression of enzymes involved in LPA synthesis and degradation [59]. A study using a three-dimensional culture model showed that LPA and S1P inhibited PC-3 cell invasion (as assessed by loss of “roundness”) and enhanced acinar morphogenesis via LPAR1 [60]. Interestingly, these investigators also found, in a screen stated to be “preliminary”, that LPAR1 expression was reduced in human prostate cancer samples as compared to normal tissues, and further reduced in metastatic prostate cancer. A similar trend was not found for S1PRs. Given the unexpected nature of these findings, further studies in additional 3-D culture models are needed. The role of LPA-induced VEGF expression in promoting lymphatic metastasis has been reviewed [61]. LPA was shown to inhibit cell death induced by docetaxel, estramustine, and mitoxantrone in PC-3 cells [62].

Considerable research is underway to develop LPA receptor antagonists, in view of the fact that GPCRs are highly “druggable” [63]. It should however be noted that LPAR1, shown to be particularly important in prostate cancer cells, is widely expressed and involved in diverse physiological processes. Thus, systemic LPAR1 antagonism carries potential off-target risks. However, this is the situation for many GPCR targets, and often the side effects are surmountable. Clinical trials of LPA antagonists are underway for various cancers [64]. A recent trial of an LPAR1 antagonist for treatment of pulmonary fibrosis indicated a safety profile conducive to phase III trials [65], along with a delayed time to disease progression [66]. Pulmonary fibrosis has been a priority for such therapy because LPA is known to play a major role in the pathology. Autotaxin inhibitors have also been under development as potential therapeutics in cancer and other diseases [67]. These inhibitors are anticipated to be less selective for specific cells and tissues than LPA antagonists, since autotaxin is a secreted enzyme that can generate paracrine LPA. There are recent examples of translational development and clinical trials of ATX inhibitors [68,69]. The latter study, in pulmonary fibrosis, was ended early due to lack of benefit. Thus, for both LPA antagonists and ATX inhibitors, drug development is still underway. With LPA showing potential for the treatment of multiple human conditions, clinical trials have progressed to phase II for treatment of diseases other than prostate cancer.

### 2.3. Eicosanoids

Eicosanoids are bioactive metabolites of arachidonic acid. Briefly, arachidonic acid is released from cellular phospholipids by the action of phospholipase A_2_ (PLA_2_) isoforms; this is the rate-limiting step in eicosanoid synthesis. Some forms of PLA_2_ are regulated by signal transduction and can be activated by receptor agonists. Arachidonic acid is converted to prostaglandin H_2_ (PGH_2_) by cyclooxygenase (COX) isoforms. PGH_2_ is then rapidly converted to a plethora of prostaglandins and thromboxanes via separate enzymatic pathways. Arachidonic acid is also be metabolized by 5-lipoxygenase (5-LOX) to 5(S)-hydroperoxy-6-trans-8,11,14-cis-eicosatetraenoic acid (5-HPETE), which is subsequently converted to leukotrienes. Multiple forms of LOX are required for the biosynthesis of lipoxins in a transcellular process that typically involves more than one cell type. The profile of eicosanoid metabolites produced within a given cell type is dependent upon its levels of expression of the various enzymes involved, and the regulation of these enzymes. Although the profiles are unique, nearly all nucleated cells have been shown to generate eicosanoids. Eicosanoids generally act locally as autocrine or paracrine mediators, although they can also be found in the circulation. Arachidonic acid metabolites have long been noted to play a variety of roles in prostate cancer, as well as in other cancers, and their enzymatic pathways and receptors present multiple therapeutic targets [70,71]. Each class of eicosanoid will be reviewed separately below.

#### 2.3.1. Prostaglandins

Prostaglandins are lipid mediators in the eicosanoid family that are prominent for their roles in pain and inflammation. Prostaglandins exert their effects by activating multiple GPCRs with varying selectivity. Of particular relevance here, PGE2 activates the GPCRs EP1-4. The pro-oncogenic effects of prostaglandin E_2_ in prostate cancer have been recognized for over 40 years, and have been previously reviewed [72,73]. PGE_1_, another endogenous E-series prostaglandin, is structurally similar to PGE_2_ and activates EP1 and EP4. However, PGE_1_ is not derived from arachidonic acid, and has distinct biological activities. The enzymatic steps involved in PGE_2_ synthesis have also been discussed in detail elsewhere [74]. PGE_2_ is one of the most prevalent eicosanoids in the body. Although PGE_2_ has a longer half-life than many other eicosanoids, it is inactivated by virtually all tissues and only persists in human circulation for 5–10 s. Nonetheless, paracrine actions of secreted PGE_2_ on adjacent cells and tissues are worthy of consideration with respect to the cancer microenvironment.

Studies examining protein expression have implicated prostaglandins in human prostate cancer progression. An early report indicated that expression of the GPCRs EP1, EP2, and EP4 was associated with prostate cancer, with EP1 appearing to be particularly important [75]. Expression of COX-2 was positively correlated with advanced patient age, higher levels of PSA, higher incidence of relapse, and worse disease overall [76]. A subsequent paper from this group showed that expression of EP4, but not EP1-3, was correlated with castration-resistant prostate cancer in human tissue samples [77].

An advantageous aspect of prostaglandin functional studies is the availability of COX inhibitors. These small molecule inhibitors, referred to as non-steroidal anti-inflammatory drugs (NSAIDs), are widely used for a variety of human conditions since they suppress the inflammatory responses mediated by prostaglandins. Use of NSAIDs was correlated with decreased risk of prostate cancer in a human population-based study [78].

Multiple published studies have demonstrated a role for PGE_2_ in prostate cancer cell growth and survival. In 2018, one group showed that COX-2 was up-regulated in highly invasive prostate cancer cells [79]. Application of PGE_2_ or over-expression of COX-2 increased activity of matripase, a membrane protease, and also increased cell invasion. Silencing of COX-2 expression had the opposite effect. In the same report, a COX-2 inhibitor decreased PC-3 cell invasion. Similarly, others showed that inhibitors of PLA_2_ or COX inhibited invasion of PC-3 and Du145 human prostate cancer cell lines through Matrigel [80]. Inhibitors of 5-lipoxygenase (LOX) did not exert this effect. Induction of COX-2 plays a role in prostate hyperplasia induced by bisphenol A, an environmental toxicant [81].

With respect to the GPCRs involved in PGE_2_ response in human prostate cancer cells, one group demonstrated that PGE_2_ activates both EP2 and EP4 receptors in PC-3 cells, and transactivates EGFR [82]. This study examined various signal transduction events, with an emphasis on those related to angiogenesis. Others showed that extracellular PGE_2_ is taken up by PC-3 cells by a prostaglandin transporter to activate intracellular EP2 receptors [83]. Responses to PGE_2_ included proliferation, migration, invasion, angiogenesis, and loss of cell adhesion. Intracellular PGE_2_ transactivates EGFR to initiate signaling events that result in upregulation of COX-2; this constitutes a positive feedback loop that maintains cell growth [84]. Another team confirmed that PGE2 enhances proliferation and migration of PC-3 cells via EP4 receptors [85]. Taken together, these reports indicate that both EP2 and EP4 contribute to PGE_2_ signaling in PC-3 cells.

With respect to the prostate tumor microenvironment, one group examined the effects of PGE_2_ on the migration of tumor-infiltrating T cells and CD11c dendritic cells into RM-1 cell tumor sites in mouse xenografts [86]. In this model, PGE2 promoted immune cell migration in a manner that impaired tumor growth. These results serve as an example of how the effects of lipid mediators on non-tumor cells in the tumor microenvironment can act in opposition to the effects on the tumor cells themselves. This is why the use of animal models is particularly important when targeting receptors that are expressed in multiple cell types.

PGE_2_ is a recognized therapeutic target in multiple types of cancer [87]. The most expeditious approach to addressing the role of PGE_2_ in human prostate cancer is to examine the effects of NSAIDs, which are widely used and have a known safety profile. In 2009, it was reported that the NSAID naproxen acted synergistically with calcitriol to suppress prostate cancer in human patients [88]. The effects of NSAIDs and other anti-inflammatory agents in prostate cancer have been examined from multiple angles, and were reviewed in 2020 with consideration of tumor immunology and effects on the tumor microenvironment [89]. Another review has addressed the effects of NSAIDs, and the roles of prostaglandins, thromboxanes, and prostanoid receptors, in a variety of cancers including prostate [90]. In addition to NSAIDs, which suppress the production of prostaglandins, small-molecule antagonists have been developed that are selective for different EP receptors including EP2 and EP4 [91,92,93,94]. These drugs have advanced to clinical trials for the treatment of multiple human disease states. Prostaglandin antagonists may be beneficial in prostate cancer therapy for their anti-inflammatory and immunosuppressive effects, as well as for their direct actions on cancer cells.

Overall, the beneficial effects of using NSAIDs to reduce PGE_2_ levels in prostate cancer patients are well established. The effects of EP receptor antagonists are rapidly being explored in the clinic.

#### 2.3.2. Thromboxanes

As an alternative to the generation of prostaglandins, COX-generated PGH_2_ can be metabolized by thromboxane synthase (TXS) to generate thromboxane A_2_ (TXA_2_). TXA_2_ is a bioactive eicosanoid best recognized for its ability to induce platelet aggregation. There are two major forms of the thromboxane GPCRs, TPα and TPβ, which are generated by alternative splicing from a single gene [95].

In 2004, one group published a study concerning the expression and function of TXS in human prostate cancer cell lines [96]. These investigators found that TXS protein was more highly expressed in advanced prostate tumors. Interestingly, TXS protein levels were reduced by COX inhibitors. A role for TXA_2_ in PC-3 cell migration on fibronectin was demonstrated using both enzyme inhibitors and TXS over-expression. A subsequent paper from the same team examined TXA_2_-initiated signal transduction (e.g., Rho activation) in PC-3 cells [97]. In this work, they showed that PC-3 cells express TPα, and that TP agonists induced cell contraction and activated the small RhoA. A Rho kinase (ROCK) inhibitor blocked agonist-induced contraction. On a related note, other investigators reported that TXA_2_ induces contraction of human prostate smooth muscle in patient-derived tissue samples via Rho- and calcium-mediated pathways [98]. TXS was expressed in glandular cells, while TP was expressed by both glandular and smooth muscle cells.

With respect to the clinical significance of these findings, several groups have analyzed data from human prostate cancer samples. In tissue slices from human patients, expression levels of TXS and TPs were elevated in malignant prostate cells as compared to nontumor cells [99]. These investigators also noted that COX-2 levels were elevated in tumor cells. The increases in COX-2 protein levels were positively associated with higher Gleason scores and pathologic stages. A later report examined the expression of TP isoforms α and β in more detail, using microarrays from human tissue samples [100]. These investigators found that expression of TPα was variable, but that higher expression of TPβ, and differences in methylation status of its promoter, were correlated with malignancy and with Gleason grade.

Turning to data from human patients, high levels of urinary TXAB_2_ (a stable metabolite of TXA_2_) have been positively associated with metastatic prostate cancer [101]. Similarly, high TXAB_2_ was associated with all-cause mortality and prostate cancer-specific mortality. Aspirin use was correlated with lower levels of urinary TXB_2_. Through its inhibitory effects on TXA_2_ -mediated platelet activation, aspirin can help prevent some cancers [102]. A separate study confirmed that TXAB_2_ levels are higher in patients with prostate cancer [103]. Interestingly, androgen deprivation therapy further increased TXAB_2_ levels in prostate cancer patients. This finding is of concern because elevated TXA_2_ presents a risk of thrombosis, and ADT increases cardiovascular risk in humans [104].

TP antagonists and TXS inhibitors have been developed for potential use in cardiovascular diseases [105]. As is the case for prostaglandin receptor antagonists, the TXA_2_ receptor antagonists have the advantage of being more cell- and tissue-selective than NSAIDs. Anti-TXA_2_ agents are being considered for the therapy of other conditions, including inflammation and cancer [95]. In regard to cancer, the anti-thrombotic effects of TXA_2_ agents could be either an advantage or a disadvantage, depending on the patient circumstances.

#### 2.3.3. Leukotrienes and Lipoxins

Leukotrienes are arachidonic acid metabolites produced by 5-LOX and not by COX. The lipoxygenase pathway has been examined with respect to prostate cancer. Major LT species are (1) LTB_4_, and (2) the cysteinyl-LTs (LTC_4_, LTD_4_, and LTE_4_); these are GPCR agonists binding to BLTs and CysLTs, respectively. LTB_4_ and its high affinity receptor BLT2 are of most interest with respect to prostate cancer.

An analysis of the expression of LOX isoforms in human tissues indicated that 5- and 12-LOX were expressed at higher levels in prostate cancer than in patients with benign prostatic hyperplasia or in normal subjects [106]. This study also showed that LOX inhibitors cause apoptosis of PC-3 cells. Another form of LOX, 15-LOX, is expressed in benign prostate tissue but is decreased in prostate tumors [107]. This enzyme is involved in the biosynthesis of lipoxins, another class of lipid mediators that plays roles in resolving inflammation [108]. A later study showed that expression of 5-LOX was expressed more highly in tumors from TRAMP mice with metastases, but this result was not seen with human tissue samples [109].

The cellular effects of leukotriene synthesis inhibitors have been reported in prostate cancer cells. An early report showed that PC-3 and LNCaP cells produce 5-HETE [110]. Inhibition of 5-LOX induced apoptosis in both cell types, while COX inhibitors did not have this effect. In the same year, another group similarly found that 5-LOX inhibitors induce apoptosis in PC-3 cells [111]. The 5-LOX inhibitor MK591 causes apoptosis in LNCaP cells [112]. The same group later reported that MK591 inhibits invasion and induces apoptosis in C4-2B human prostate cancer cells [113]. Treatment of PC-3 and LNCaP cells with a 5-LOX inhibitor resulted in higher levels of paxillin and E-cadherin, and suppressed motility [109]. Another report showed that celecoxib, a COX-2 inhibitor, inhibited PC-3 cell survival via its dual ability to inhibit 5-LOX rather than via COX-2 [114]. The inhibitory effects were more prominent using a 5-LOX inhibitor, and were reversed by LTB_4_.

Prostate cancer cells express GPCRs that are activated by leukotrienes. PC-3 cells express BLT2, the receptor for LTB_4_ [115]. In this study, there was an increase in BLT2 expression following detachment of PC-3 cells from substrate. The team found that either a BLT2 antagonist or LTB_4_ knockdown increased apoptotic cell death occurring after cell detachment (“anoikis”), and that over-expression of BLT2 in the normal prostate cell line PWR-1E conferred resistance to anoikis.

Lipoxins have not been fully investigated in prostate cancer. As recently reviewed, the lipoxin LXA_4_ may promote prostate cancer cell growth via activation of the GPCR ALX/FPR2 [104]. L4A_4_ is produced in a complex transcellular biosynthetic pathway (e.g., between neutrophils and epithelial cells) involving 15-LOX and 5-LOX.

Overall, much work remains to be done to define the roles of leukotrienes and lipoxins in prostate cancer. As recently reviewed, the findings to date indicate that 5- and 12-LOX are involved in promoting prostate cancer, while the overall role of 15-LOX-2 is inhibitory [116]. The existing data also indicate that LTB_4_ is of particular interest with respect to prostate cancer. LOX inhibitors have received the most attention on the therapeutic side to date, but LOX receptor antagonists have been developed for use in non-cancer disease states and are available for further testing. The roles of lipoxins bear further scrutiny. Progress in using lipoxins as therapeutic targets for drug development, in various diseases, was recently presented [108].

### 2.4. Cannabinoids

Cannabinoids from plant sources (phytocannabinoids, e.g., from *Cannabis sativa*) and mammals (endocannabinoids) are biosynthesized by different routes. The endocannabinoids, 2-arachidonoyl-glycerol (2-AG) and *N*-arachidonoyl-ethanolamine (AEA; anandamide) activate the GPCR cannabinoid receptors CB_1_ and CB_2_. Endocannabinoids are technically eicosanoids, since some are derived from arachidonic acid, but they are considered as a separate class. The multiple enzymatic pathways involved in endocannabinoid biosynthesis and metabolism have been reviewed [117]. Cannabinoids are available in multiple forms as phytochemicals and as synthetic derivatives. Cannabidiol (CBD) is a phytocannabinoid that is neither an eicosanoid nor technically a lipid mediator, but is included in the discussion below because it is a potential therapeutic agent. CBD binds with low affinity to CB receptors, and with higher affinity to TRPV1 channels.

An early study in this area addressed the expression of cannabinoid receptors in a panel of human prostate cancer cell lines and primary human prostate epithelial cells [118]. The results indicated that both CB_1_ and CB_2_ are more highly expressed in prostate cancer cell lines than in primary or “normal” prostate epithelial cells. A mixed CB_1_/CB_2_ agonist inhibited proliferation of LNCaP cells but not normal prostate epithelial cells; the response was blocked by either a CB_1_ or CB_2_ antagonist. Further observations included that the antagonists induced apoptosis and decreased expression of androgen receptor in LNCaP cells. In a subsequent report, the same group examined the signal transduction events involved in CB agonist-induced inhibition of cell growth and induction of apoptosis [119]. These early findings have been reviewed [120].

More recently, the effects of cannabinoids on cancer-associated fibroblasts in prostate cancer were investigated [121]. The authors found that a CB agonist decreased migration of patient-derived fibroblasts and interfered with other aspects of fibroblast activation. These responses were mediated mainly by CB_2_. The authors also confirmed that a CB agonist inhibits proliferation of prostate cancer cells but not normal prostate epithelial cells.

The cell culture results led to studies examining the effects of CB agonists on prostate tumor growth (PC-3 and Du145 cell xenografts) in rodent models. These studies have been reviewed, with the overall conclusion that the CB agonists decreased tumor growth [122].

The effects of the phytocannabinoid cannabidiol (CBD) on prostate cancer cells have been explored. In one very comprehensive study, the effects of a panel of agonists and antagonists for CB receptors, TRPA channels, and TRPV1 channels were tested in PC-3, Du145, 22RV1, and LNCaP cells [123]. Of the compounds tested, CBD was particularly potent and efficacious in inhibiting viability of LNCaP and Du145 cells. The effects of CBD were independent of CB receptors; TRPM8 was implicated in some of the responses analyzed. In rodent xenografts, CBD inhibited the growth of LNCaP tumors, but not Du145 tumors. The same report included data concerning mechanisms involved in CBD-induced apoptosis. More recently, another group revisited the effects of CBD on PC-3, Du145, and LNCaP cells [124]. They confirmed anti-proliferative effects of CBD on these cells, but interestingly found that the “normal” cell lines PWR-1E and RWPE-1 were even more sensitive to CBD. The effects of CBD were not blocked by CB antagonists or TRPV1 channel blockers. The investigators also found that CBD reduced invasion of PC-3 cells in a Matrigel transwell assay. In a separate study, researchers found that CBD inhibited PC-3 cell proliferation and induced apoptosis [125]. The signal transduction pathways involved (e.g., Rho, NFκB, NRF2) were also examined in this study.

Given the relatively widespread use of cannabis products, it is not surprising that their effects have been examined in prostate cancer patients. For example, in one study patients were treated with cannabis oil alone, chemotherapy alone, or a combination of cannabis oil and chemotherapy [126]. The cannabis oil contained both CBD and the psychoactive constituent tetrahydrocannabinol (THC). The results showed that cannabis oil improved pain control and quality of life, with some indicators of a positive tumor response. However, current policy statements from medical societies emphasize that CBD has not been proven to improve outcomes in prostate cancer patients. This is a situation where the availability of herbal products, which contain multiple constituents, is making it difficult to determine whether any individual cannabinoids have beneficial effects. A more pharmacologically focused research effort would be beneficial.

Overall, additional work is needed to define the potential therapeutic actions of CB receptor agonists in prostate cancer. The inhibitory effects of CBD on prostate tumors are provocative, although the mechanism of action remains to be delineated.

### 2.5. Free Fatty Acids

Fatty acids hold a unique place in the pantheon of lipid mediators. They are dietary constituents, phospholipid metabolites, and also players in intermediary metabolism. They exist in a plentitude of species in human plasma and cells. The species that have been most studied with respect to prostate cancer are oleic acid, docosahexaenoic acid (DHA), and eicosapentaenoic acid (EPA). Arachidonic acid, discussed earlier, is also a free fatty acid but is so rapidly metabolized that it is not generally considered to be a mediator in its own right. The omega-3 fatty acids (e.g., DHA and EPA) have long been of interest with respect to their health benefits, particularly with respect to metabolic disease and inflammation. EPA and DHA are largely of dietary origin, and not endogenous, although they can be generated in humans from dietary alpha-linoleic acid. The involvement of free fatty acids in cellular signal transduction has been difficult to delineate. A longstanding paradigm was that the omega-3 fatty acids act largely by reducing the production of pro-inflammatory eicosanoids, essentially by replacing a portion of the arachidonic acid in cellular phospholipids. A breakthrough occurred when a family of orphan GPCR receptors were found to be activated by free fatty acids. These receptors, termed free fatty acid receptors 1–4 (FFAR1–4), are coupled to the usual G-protein-mediated response pathways and also to arrestin-mediated responses [127]. The four FFARs have varying selectivity for fatty acids [128]. Importantly, omega-3 fatty acids act as agonists for FFA2 and FFA4. The roles of FFARs in cancer cells, and of omega-3 fatty acids in prostate cancer, have been previously reviewed [129,130,131]. The literature concerning the effects of a diet rich in omega-3 fatty acids has been the topic of numerous reviews and is somewhat controversial; this is not surprising given the uncertainties involved in interpreting the effects of a dietary constituent that also acts as an energy source in the human body.

The effects of free fatty acids on prostate cancer cells have been investigated. In 2015, our group showed that the omega-3 fatty acids EPA and DHA, as well as pharmacologic agonists for FFAR4, inhibited proliferation of PC-3 and Du145 cells [44]. Signaling responses to LPA that were inhibited by FFAR agonists included Erk activation, FAK phosphorylation, p70 S6K activation, and induction of the matricellular protein CCN1. Importantly, the effects of the omega-3 fatty acids were mimicked by TUG-891, a selective pharmacologic FFAR4 agonist that (unlike EPA and DHA) is not incorporated into cellular phospholipid. Knockdown experiments indicated that FFAR4 was responsible for the growth inhibitory effect. This was an unexpected observation, since FFAR4 was known to activate typical GPCR-mediated pathways that are generally involved in cell proliferation. In subsequent work, we further explored the mechanism, showing that activation of FFA1 interfered broadly with LPA and EGF responses in these cells and that arrestin-3 was involved [41]. In an intriguing mechanistic investigation, the Garcia-Sainz research group used engineered HEK293 cells to show that FFAR4 agonists cause desensitization of the LPA receptor that is independent of receptor internalization [132]. Intriguingly, in the same study the investigators demonstrated that there are physical interactions between FFAR4 and LPA1. Thus, the LPA1-FFAR4 interaction appears to represent a previously unrecognized type of receptor cross-talk. The roles of FFAR4 in cancer cells, including prostate cancer cells, have been previously reviewed [133].

In 2016, it was reported that oleic acid, which activates FFA1 and FFA4, enhances proliferation of PC-3 and Du145 cells [134]. These investigators attributed the effects to the FFA1 receptor, and showed that FFA1 was over-expressed in malignant prostate tissue as compared to benign prostatic hyperplasia tissue. In 2018, another group reported that FFA1 and FFA4 mediated DHA-induced apoptosis of PC-3 and Du145 cell lines [135]. The investigators attributed the response to DHA-induced activation of the Hippo pathway and YAP phosphorylation. The YAP pathway is known to induce CCN1, consistent with our results showing that LPA increases CCN1 in prostate cancer cells [42].

GPCR-mediated effects of fatty acids are not the only mechanism that has been explored in prostate cancer. A more recent review focusses on non-GPCR mechanisms involved in the effects of DHA on prostate cancer cells [136]. Omega-3 fatty acids also exert indirect effects in prostate cancer via the tumor microenvironment and on immune cells such as macrophages [137]; some of these effects are mediated by GPCRs.

Despite the many reviews on omega-3 fatty acids and prostate cancer, there are relatively few original research reports concerning FFAR-mediated actions in prostate cancer cells. The roles of arrestin proteins in the actions of omega-3 fatty acids and other lipid mediators binding to GPCRs have not been fully explored, even though they present new therapeutic possibilities [138]. The development of FFAR synthetic ligands, both orthosteric and allosteric, provides new routes for therapeutic interventions that are pharmacologic rather than dietary [139]. Structural insights into FFARs and their interactions with other proteins will be helpful in this regard [140].

## 3. Conclusions

**Figure 1:**
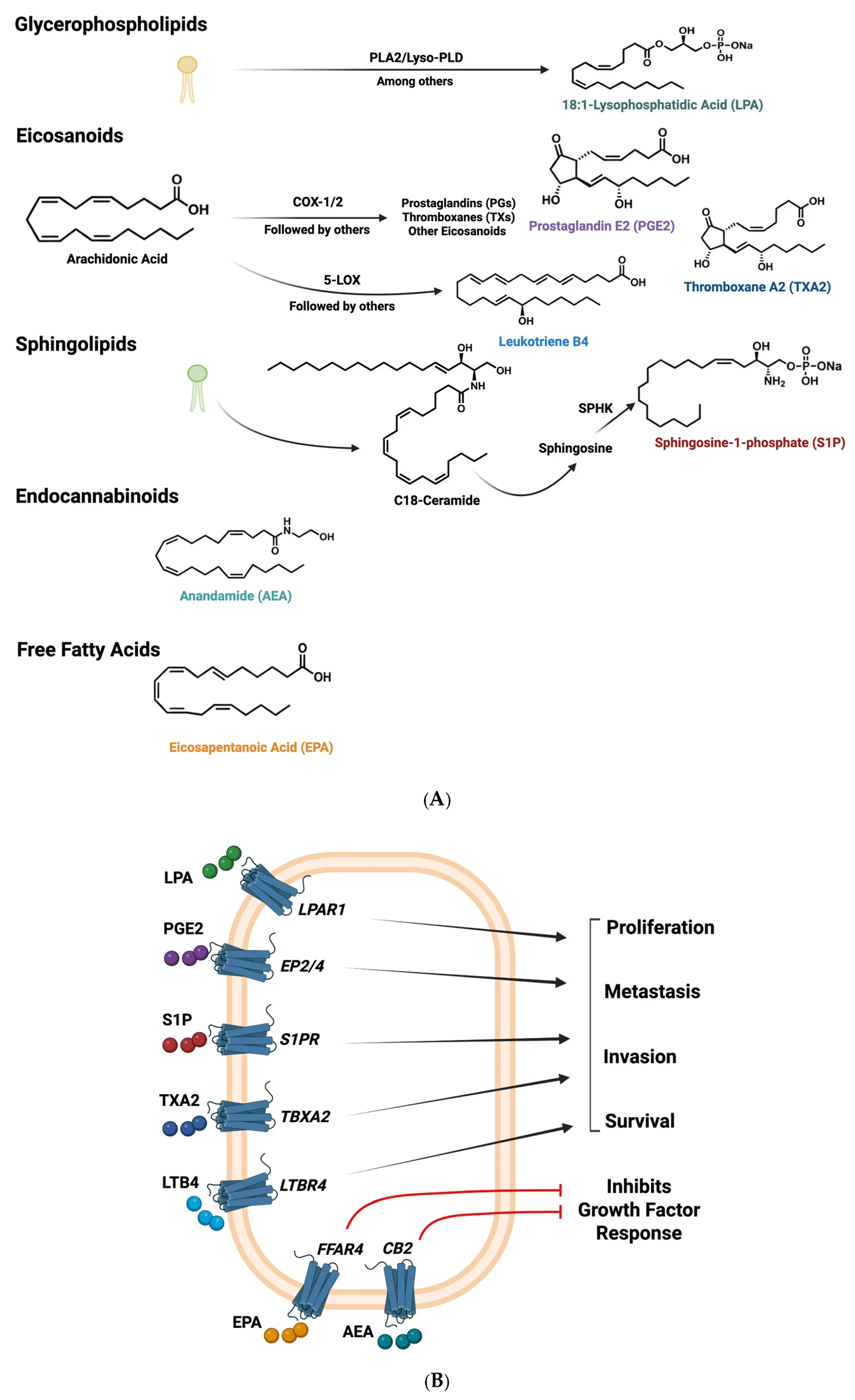
Lipid mediators and their GPCRs. (**A**) Chemical structures of the lipid mediators discussed in the text, with the major enzymatic pathways involved in their synthesis. (**B**) Depiction of a prostate cancer cell expressing multiple GPCRs that are activated by the lipid mediators discussed in the text and depicted in panel (**A**). The overall effects mediated by the receptors are indicated by the arrows.

In summary, this review has briefly presented research concerning the effects of major classes of lipid mediators on prostate cancer cells. There are two tables summarizing the findings discussed herein. The lipid mediators, their receptors, and pharmacologic agents are listed in Table 1. In Table 2, enzymes involved in mediator synthesis are listed, along with the net effects of the mediators on prostate cancer cells. Figure 1A presents the chemical structures of the lipid mediators discussed. Figure 1B depicts the receptors for each type of ligand, and whether the response generally promotes or inhibits proliferation in prostate cancer cells. In almost all cases, the effects of these small-molecule mediators are mediated by GPCRs. As genomic approaches and GPCR structural biology advance, our understanding of how GPCRs recognize their ligands and activate downstream pathways is rapidly expanding. This has led to an increased appreciation of the potential value of GPCRs as therapeutic targets in cancer [141,142].

To date, the most extensive work establishing potential therapeutic targets for prostate cancer has been done for SPHK1, S1PR1/3, LPAR1, COX1/2, EP2/4, TPβ, 5-LOX, BLT2, CB_1_/CB_2,_ and FFA4. These candidate targets cannot easily be ranked relative to each other, as some of them have overlapping effects (e.g., COX inhibitors and EP antagonists; SPHK inhibitors and S1PR antagonists), and can potentially act in a synergistic manner (e.g., LPAR antagonists and FFAR agonists). The investigation of dual targeting, using combinations of targets, has not yet been fully considered. For many targets, suitable therapeutic agents are still under development. It is likely that the impact of these therapies will vary between prostate cancer patients, depending on the profile of expression of the relevant receptors in a given tumor.

There were several examples provided herein of studies examining whether strategies targeting lipid mediators can enhance sensitivity to androgen deprivation or chemotherapy. This is a standard approach used in pre-clinical cancer investigations. While positive effects of drug combinations were observed in some studies [5,11,12,17,18,19,28], especially for agents targeting S1P, it should be noted that any strategy that decreases cancer cell proliferation and/or survival is likely to enhance sensitivity to other anti-cancer treatments. A comprehensive approach to combining the two strategies, with respect to lipid mediators, has not yet emerged since in most cases clinical trials have not progressed to that point.

Most of the studies summarized herein, concerning a variety of lipid mediators, utilize the PC-3 cell line as the primary cell culture model. PC-3 is a “tried and true” model of an androgen-resistant prostate cancer cell line, and the use of a single cell line is helpful in terms of comparison and consistency. However, the use of additional cell lines, as well as patient-derived prostate cancer tissue, will be beneficial as studies in this field moves further into the translation phase. It is too early to predict whether lipid mediators are more important in advanced prostate cancer than in early stages of progression.

Effects of lipid mediators on the tumor microenvironment have, to date, been addressed in only a few reports regarding prostate cancer [15,86,89,121,137]. Further examination of this topic is extremely important, given the fact that receptors for the lipid mediators are widely expressed in various cell types, with the different portfolios of expression of specific receptors between cells lending nuance to the effects of the mediators.

In view of the fact that some of the mediators have been examined for more than 20 years, and that inhibitors of their production and/or action are available in many cases, one might have expected to see more progress with respect to translation of the findings. In general, the roles of GPCRs as cancer targets has been slow to evolve. In part, this is due to the fact that GPCRs do not typically present as targets for a “magic bullet” that will kill cancer cells. Rather, GPCR ligands modulate cellular signaling in ways that can reduce proliferation and inhibit cell migration. They can enhance the effects of chemotherapeutic agents in advanced tumors. However, it is possible that GPCR pharmaceutics will play more important roles in suppressing early-stage cancers, although conventional drug testing in prostate cancer generally focusses on androgen-independent tumors since the therapeutic needs are so urgent. As reviewed here, lipid mediators are already the targets of drug development, a time-consuming process. Clinical trials are gradually emerging, after suitable lead compounds have been generated. However, the results of these trials are not yet definitive and it will be necessary to test multiple compounds.

An additional consideration is that the GPCRs of interest are widely expressed and play important roles in normal tissues. They are sometimes over-expressed in cancer cells, but are not exclusive to them. Nonetheless, the non-cancer side effects of GPCR pharmaceuticals, for the receptors discussed here, are generally mild in nature as compared to those of chemotherapeutic agents.

In this era of multifaceted approaches to cancer, and the use of drug combinations to ameliorate the side effects of therapy with cytotoxic drugs, there is certainly much to consider in this field. Drugs that modulate the levels or activities of lipid mediators may prove useful in balancing overall patient health and well-being with reduced cancer recurrence. In the right context and the right patient, drugs targeting lipid mediators may be helpful in maintaining quality of life while discouraging cancer cell proliferation. There are many exciting avenues to pursue in this field, both mechanistically and clinically. To quote American educator and journalist Margaret Fuller (1810–1850), “If you have knowledge, let others light their candles at it” [143].

## Figures and Tables

**Table 1:** Major classes of lipid mediators active in prostate cancer cells.

Mediator Class	Key Representative(s)	Receptors	Pharmacologic Agents
Glycerophospholipids	Lysophosphatidic Acid (LPA)	LPA 1–6	ATX inhibitors LPAR1/3 antagonists
Sphingolipids	Sphingosine 1-Phosphate (S1P), Ceramide	SIP 1–5	SphK1/2 inhibitors S1P1/3 antagonists
Eicosanoids (PGs)	Prostaglandin E_2_ (PGE_2_)	EP1, EP2, EP4	COX inhibitors (NSAIDs) EP2/EP4 antagonists
Eicosanoids (TXs)	Thromboxane A_2_ (TXA_2_)	TPα, TPβ	TS inhibitors TP antagonists
Eicosanoids (LTs)	Leukotriene B_4_ (LTB_4_), Lipoxin A_4_	BLT1, BLT2, ALX/FPR2	5-LOX inhibitors BLT2 antagonists ALX/FPR2 antagonists
Cannabinoids	AEA, 2-AG, Cannabidiol (CBD)	CB1, CB2, TRPV1, TRPM8	Synthetic CB_2_ agonists Phytocannabinoids
Free Fatty Acids	EPA, DHA, Oleic Acid	FFA1, FFA4	FFA4 agonists

**Table 2:** Biosynthesis and cellular actions of lipid mediators in prostate cancer.

Mediator	Key Biosynthetic Enzymes	Effect on Prostate Cancer Proliferation/Survival
Sphingosine 1-phosphate (S1P)	Sphingosine kinase 1 (SPHK1)	stimulates
Lysophosphatidic acid (LPA)	PLA_2_, PLD, lyso-PLD (ATX)	stimulates
Prostaglandin E_2_ (PGE_2_)	PLA_2_, COX	stimulates
Thromboxane A_2_ (TXA_2_)	PLA_2_, Thromboxane synthase (TS)	stimulates
Leukotriene B_4_ (LTB_4_)	PLA_2_, 5-LOX	stimulates
Lipoxin A_4_ (LXA_4_)	PLA_2_, 15-LOX	stimulates
Endocannabinoids	[multiple pathways]	inhibits
Omega-3 fatty acids	[largely dietary]	inhibits

## Data Availability

No new data were generated for this review article.

## Abbreviations

The following abbreviations are used in this manuscript:

3LS	three-lipid signature
AC	acid ceramidase
ADT	Androgen deprivation therapy
ATX	autotaxin
CBD	cannabidiol
COX	cyclooxygenase
DHA	docosahexaenoic acid
EGF	epidermal growth factor
EPA	eicosapentaenoic acid
FFA	free fatty acid
GPCR	G protein-coupled receptor
LOX	lipoxygenase
LPA	lysophosphatidic acid
LT	leukotriene
NSAID	non-steroidal anti-inflammatory drug (COX inhibitor)
PG	prostaglandin
S1P	sphingosine-1-phosphate
SPHK	sphingosine kinase
SPL	sphingosine lyase
TX	thromboxane
TXS	thromboxane synthase
